# Isolation and Characterization of Lactic Acid Bacteria from an Italian Traditional Raw Milk Cheese: Probiotic Properties and Technological Performance of Selected Strains

**DOI:** 10.3390/microorganisms13061368

**Published:** 2025-06-12

**Authors:** Marianna Roselli, Federica Colafranceschi, Valentina Cipriani, Alessandra Valle, Paola Zinno, Barbara Guantario, Emily Schifano, Daniela Uccelletti, Chiara Devirgiliis

**Affiliations:** 1 Research Centre for Food and Nutrition, CREA (Consiglio per la ricerca in agricoltura e l’analisi dell’economia agraria), Via Ardeatina 546, 00178 Rome, Italy; colafranceschif@yahoo.it (F.C.); v.cipriani@policlinicocampus.it (V.C.); ale.valle3@stud.uniroma3.it (A.V.); barbara.guantario@crea.gov.it (B.G.); 2 Institute for the Animal Production System in the Mediterranean Environment (ISPAAM), National Research Council, Piazzale Enrico Fermi 1, 80055 Portici, Italy; paola.zinno@cnr.it; 3 Department of Biology and Biotechnologies “C. Darwin”, Sapienza University of Rome, Piazzale Aldo Moro, 00185 Rome, Italy; emily.schifano@uniroma1.it (E.S.); daniela.uccelletti@uniroma1.it (D.U.)

**Keywords:** food microbiota, food quality, fermented dairy products, food–gut axis, nematode, antagonistic activity, lactobacilli

## Abstract

The increasing interest in fermented foods stems from their health benefits, mediated by foodborne microorganisms. This study aimed to characterize the fermentative microbiota of Pecorino di Picinisco, a traditional Italian cheese made from ovine raw milk, and to evaluate the probiotic and technological potential of selected lactic acid bacteria strains. Three strains representative of the different species found (*Lactococcus lactis*, *Lactiplantibacillus plantarum* and *Latilactobacillus curvatus*) were chosen and analyzed. All three strains were able to adhere to human intestinal Caco-2 cells, were resistant to simulated in vitro digestion and significantly prolonged the lifespan of *Caenorhabditis elegans*, used as a simplified *in vivo* model, with respect to the commercial probiotic strain *Lacticaseibacillus rhamnosus* GG. The *L. plantarum* Pic37.4 strain was particularly promising; therefore, its cell-free supernatant was employed to evaluate the antimicrobial activity against indicator strains of foodborne and intestinal pathogens or spoilage bacteria. The results demonstrated the effectiveness of the supernatant against all strains tested, with the strongest effect on the intestinal pathogen enterotoxigenic *Escherichia coli* K88. In addition, the inhibitory effect on pathogen adhesion to intestinal mucosa was investigated on Caco-2 cells, resulting in a significant reduction in adhesion mediated by the *L. plantarum* Pic37.4 supernatant. The antimicrobial properties of the *L. plantarum* strain were confirmed *in vivo* in *C. elegans*. These promising results lay the ground for further investigations aimed at substantiating the probiotic and technological potential of the *L. plantarum* Pic37.4 investigated in this work.

## 1. Introduction

### 1.1. Background on Traditional Raw Milk Cheeses and Importance of Indigenous LAB

In recent decades, interest in fermented foods has risen, especially due to the increased awareness of the presence of microorganisms with potential beneficial activities relevant to human health [1,2,3]. Fermented foods include a plethora of different products mostly from dairy, meat and vegetable sources, characterized by various production technologies and consumption frequencies, often reflecting local resources and dietary profiles [4,5]. Differently from industrial products, obtained through defined starter cultures, traditional products are characterized by spontaneous fermentation, committed to indigenous microbes present in the raw material or in the manufacturing environment, representing highly diverse and complex, still largely uncharacterized, microbiota communities [6]. Not all the foods and beverages obtained through fermentation contain live microbes when consumed, due to different processing steps, such as pasteurization, baking, smoking or filtering. Among fermented foods, dairy products, frequently unprocessed after fermentation, represent one of the major sources of foodborne microbes ingested upon consumption [7,8,9], some of which may overcome the gastrointestinal barriers (low pH, bile salts, digestive enzymes) and reach the gut, often aided by the food matrix that protects them during this journey [10,11]. Traditional fermented dairy products, therefore, are emerging as interesting delivery vehicles of novel probiotic strains, the majority of which are lactic acid bacteria (LAB), mostly belonging to the former *Lactobacillus* and other related genera. Indeed, although the microbes in fermented foods cannot, by definition, be considered probiotic, many of them are evolutionarily highly related to probiotic organisms and often share the same molecular mechanisms underlying their health-promoting activities, as demonstrated in several model systems [12,13]. Among the different experimental models, *Caenorhabditis elegans*, a tiny soil nematode eating bacteria, has become a popular *in vivo* system for exploring how probiotics interact with hosts through the study of lifespan extension. This is due to numerous advantages, such as transparency, short life cycle, ease of cultivation and presence of many molecular pathways that regulate its development, oxidative stress response, metabolism, and host defense mechanisms, many of which, such as insulin/IGF-1 signaling (IIS) and the p38 MAPK pathway, are conserved in more complex organisms [14].

Moreover, uncharacterized foodborne LAB strains may also be promising from a technological point of view, especially concerning shelf-life extension, which can be achieved through counteracting the growth of pathogenic and spoilage microbes, therefore improving food safety and quality [15,16]. The preservative ability of LAB in foods is attributed to the production of many different antimicrobial molecules, such as peptidic or proteinaceous bacteriocins; organic acids (butyric, acetic and lactic acids); and other inhibitory compounds (diacetyl, hydrogen peroxide, acetaldehyde, acetoin, reuterin and reutericyclin) [17]. Consequently, LAB have recently emerged as valid and safe alternatives to chemical food preservatives, and a significant area of research is now focused on their use as bioprotective cultures [18,19].

### 1.2. Rationale for Screening Both Probiotic and Technological Traits in LAB Isolates

For all the above-mentioned aspects, traditional fermented foods can be relevant as a source of novel foodborne strains with interesting probiotic and technological features [20]. Screening LAB isolates for both probiotic and technological traits is crucial for their successful application in several food sector contexts. This dual approach stems from the understanding that while a bacterial strain might possess significant health-promoting (probiotic) properties, it should also be able to perform effectively within a food matrix or industrial process (technological traits). By screening for both sets of traits concurrently, multifunctional LAB strains that not only deliver significant health benefits but also contribute positively to the quality, safety, and sensory attributes of the final product can be identified. This integrated selection strategy ensures that potential probiotic candidates are not only biologically effective but also industrially viable, leading to the development of novel functional foods.

Therefore, deepening our knowledge of the microbial ecology of fermented foods will allow us not only to safeguard traditional methods but also to leverage the potential of traditional fermentation in modern food production and biotechnology, providing valuable insights into food innovation, sustainability, and health.

The present study aimed to characterize the fermentative microbiota of Pecorino di Picinisco, a traditional Italian Protected Designation of Origin (PDO) cheese made from raw ovine milk, and to assess probiotic properties and technological performance of selected LAB strains. To this purpose, through the combination of *in vitro* and *in vivo* models, the strains were evaluated for safety, adhesion to human intestinal epithelium, gastrointestinal resistance, lifespan extension in *Caenorhabditis elegans*, antimicrobial activity, and inhibition of pathogen adhesion to intestinal human cells.

## 2. Materials and Methods

### 2.1. Bacterial Strains and Growth Conditions

The LAB strains described in this work and the reference probiotic strain *Lacticaseibacillus rhamnosus* GG ATCC 53103 (LGG) were grown in De Man Rogosa Sharpe (MRS) medium for 24–48 h at 37 °C under anaerobic conditions obtained with Thermo Scientific™ Oxoid AnaeroGen gas-generating sachets (Fischer Scientific Italia, Segrate, MI, Italy). The intestinal pathogen enterotoxigenic *Escherichia coli* K88 strain (ETEC, O149:K88ac, provided by the Lombardy and Emilia Romagna Experimental Zootechnic Institute, Reggio Emilia, Italy) and *E. coli* OP50 strain (used to feed nematodes) were grown in Luria–Bertani (LB) broth at 37 °C. The foodborne pathogens *Salmonella enterica* serovar Typhimurium LT2 (DSMZ 18522) and *Listeria monocytogenes* OH (provided by the CREA Research Centre for Animal Production and Aquaculture, Lodi, MI, Italy) were grown in Tryptone Soy Broth (TSB) with 0.5% yeast extract (YE) at 37 and 30 °C, respectively. The spoilage *Pseudomonas putida* WCS358 and KT2440 strains, kindly provided by Prof. Livia Leoni of Roma Tre University (Rome, Italy), were grown in TSB with 0.5% YE at 30 °C. All media and supplements were provided by Oxoid, unless otherwise stated.

### 2.2. Antibiotic Susceptibility Tests

Antibiotic susceptibility tests were performed by pouring overnight bacterial cultures in MRS soft agar on plates containing the following antibiotic disks: ampicillin (10 µg), erythromycin (15 µg), tetracycline (30 µg), vancomycin (30 µg), kanamycin (64 µg), streptomycin (32 µg and 64 µg), gentamicin (30 µg), clindamycin (10 µg) and chloramphenicol (30 µg). All antibiotics, chosen according to the EFSA guidelines [21], were from Oxoid, except for vancomycin and streptomycin, which were from Liofilchem (Teramo, Italy). After 48 h incubation, the presence and diameter of the inhibition halos around the antibiotic disks were assessed, assuming a diameter > 1 cm as indicative of susceptibility, a diameter ≤ 1 cm as indicative of intermediate susceptibility, while the absence indicated resistance.

### 2.3. Tolerance to Gastrointestinal Conditions

An *in vitro* digestion simulation test according to Vizoso Pinto et al. [22], with modifications, was performed. Overnight cultures of LAB strains were inoculated and incubated for 16 h until early stationary phase, representing the transition point from the exponential growth to the beginning of the stationary phase, characterized by a plateau of the growth curve. Such a time point was identified based on the growth curves previously set up in preliminary experiments performed on each strain. Bacterial suspensions were centrifuged at 5000 rpm for 15 min at 4 °C and diluted 1:1 (*v*:*v*) in Simulated Salivary Juice. An aliquot of the samples was serially diluted in 0.9% NaCl and plated on MRS agar (initial time, Ti). The rest of the samples were added with 100 mg/L lysozyme and incubated for 5 min at 37 °C with gentle shaking. The samples were then diluted 3:5 (*v*:*v*) in Simulated Gastric Juice. Three g/L porcine pepsin was added, and samples were incubated for 1 h. Finally, samples were diluted 1:4 (*v*:*v*) in Simulated Pancreatic Juice, added with 0.5% bovine bile extract and 0.1% porcine pancreatin, and incubated for 3 h. All enzymes were from Merck (Darmstadt, Germany). At the end of incubations, samples were serially diluted and plated on MRS agar (final time, Tf). Plates were incubated for 48 h, then colonies from Ti and Tf were counted to calculate the survival capacity of the different strains along the digestive tract. Survival capacity was calculated as the percentage of 1– [(log CFU/mL Ti − log CFU/mL Tf)/log CFU/mL Ti], where CFU/mL Tf represented the total viable counts for each strain at the final time point of incubation in SPJ, and CFU/mL Ti represented the total viable counts at the initial time point.

### 2.4. C. elegans Strain and Growth Conditions

The wild-type *C. elegans* strain, Bristol N2, was grown at 16 °C on Nematode Growth Medium (NGM) plates covered by a layer of *E. coli* OP50, LGG, or LAB strains. LAB strains were routinely grown in MRS medium for 24 h at 37 °C under anaerobic conditions, while OP50 was grown in LB broth at 37 °C overnight. NGM was prepared as previously reported [23]. The reagents were purchased from Difco Laboratories (Detroit, MI, USA).

### 2.5. C. elegans Lifespan Assay

For lifespan assays, synchronized adult N2 worms were allowed to lay embryos for 8 h on NGM plates seeded with the different bacterial strains and were subsequently removed. Bacterial lawns were prepared as described in [24]. The assay started when the progeny reached reproductive maturity (t₀). Nematodes were transferred daily to fresh plates with newly seeded bacterial lawns and monitored for survival. Worms were scored as dead when they failed to respond to gentle touch with a platinum wire. At least 80 nematodes per condition were included in each experiment.

### 2.6. Preparation of L. plantarum Pic37.4 Cell Free Supernatant

Overnight culture of *L. plantarum* Pic37.4 was inoculated in fresh MRS and incubated for 16 h, until early stationary phase, as described above. The culture was then centrifuged at 9500 rpm for 10 min at 4 °C, and the supernatant was collected and filtered through 0.22 µm pores to remove any residual bacterial cells. The filtered cell-free supernatant (CFS) was divided into two aliquots: the first was treated with NaOH to reach pH 6.5 (neutralized CFS, CFS (N)) and the second was left as it was and brought to the same volume by adding MRS medium (CFS). The two preparations were aliquoted and stored at −20 °C.

### 2.7. In Vitro Antimicrobial Activity of Chloroform-Inactivated Cells or CFS of L. plantarum Pic37.4 Against Pathogen and Spoilage Bacterial Strains

Antimicrobial activity of *L. plantarum* Pic37.4 was tested on the indicator strains ETEC K88, *S.* Typhimurium LT2, *L. monocytogenes* OH, *P. putida* WCS358 and *P. putida* KT2440, each freshly inoculated 1:100 after overnight growth and used at exponential phase (2–4 h growth).

Concerning the agar double-layer diffusion method, performed according to Damaceno et al. [25], 3 µL of *L. plantarum* Pic37.4 overnight culture were spotted onto MRS agar and incubated for 24 h. After incubation, bacterial cells were killed by chloroform exposure for 30 min. To confirm that the chloroform had no residual inhibitory effect, a chloroform-treated negative control plate (absence of *L. plantarum* Pic37.4) was included, and uniform growth of each indicator strain was observed after incubation at the specific growth temperature. Plates were then overlaid with appropriate soft agar medium containing 1% (*v*/*v*) of each indicator strain grown as described above and incubated for 24 h. The presence of a growth inhibition halo around each spot was indicative of antagonist activity, and the corresponding diameter was measured (cm).

The antimicrobial activity of *L. plantarum* Pic37.4 CFS was instead determined by well diffusion (Supplementary Methods) and liquid broth assays. For the liquid broth assay, each indicator strain, grown as described above, was dispensed into 96-well plates at a volume of 180 or 150 µL per well. Then 20 or 50 µL of *L. plantarum* Pic37.4 CFS or CFS (N) were added to each well. As controls, equivalent volumes of MRS medium and MRS adjusted to pH 4 (acidified MRS, MRS (A)), corresponding to the pH of *L. plantarum* Pic37.4 CFS, were used. Bacterial growth was monitored by recording the OD_600_ for 22 h at 1 h intervals using an automated plate reader (INFINITE M200, Tecan, Milan, Italy). The OD_600_ values were normalized with respect to the medium alone.

### 2.8. In Vivo Antimicrobial Activity of L. plantarum Pic37.4 Against Pathogen Indicator Strains

For *C. elegans* infection assays, 3.5 cm NGM plates were prepared by spreading 30 μL of a bacterial suspension containing *L. plantarum* Pic37.4 or LGG mixed with either *S.* Typhimurium LT2, *L. monocytogenes* OH or ETEC K88 in a 1:1 ratio (1 × 10⁸ CFU/mL each). Plates seeded with pathogen alone served as controls. During the assay, 80 synchronized worms per condition were transferred daily to fresh plates with newly seeded bacterial cultures and monitored for survival. Infections were carried out at 25 °C.

### 2.9. Intestinal Caco-2 Cell Culture Conditions

Caco-2 cells, obtained from INSERM (Paris, France), were routinely sub-cultured at 50–60% density, according to Natoli et al. [26] and maintained at 37 °C in a 95% air/5% CO_2_ atmosphere at 90% relative humidity in complete Dulbecco’s Modified Eagle Medium (DMEM) containing 25 mM glucose, 3.7 g/L NaHCO_3_, 4 mM L-glutamine, 1% non-essential amino acids, 1 × 10^5^ U/L penicillin, 100 mg/L streptomycin, and 10% heat-inactivated fetal bovine serum (FBS, Euroclone, Milan, Italy). Cell culture media and reagents were from Corning (Milan, Italy), unless otherwise stated.

### 2.10. LAB Adhesion Assay to Caco-2 Cells

Caco-2 cells were seeded in 24-well plates and left, after confluency (1 × 10^6^ cells/well), for 14–17 days to allow complete differentiation, with medium change every other day [27]. Complete DMEM was replaced with antibiotic- and FBS-free DMEM 16 h before the assay. On the day of the assay, overnight bacterial cultures of *L. curvatus* Pic37.1, *Lc. lactis* Pic37.3, *L. plantarum* Pic37.4 and LGG were diluted 1:10 in MRS medium and grown for 5 h, 3 h, 4 h and 3 h, respectively, to reach exponential growth phase (approximately 1 × 10^8^ CFU/mL), according to their respective growth curves previously set up in preliminary experiments.

After monitoring the OD_600_, appropriate amounts of bacterial cells were harvested by centrifugation, resuspended in antibiotic- and FBS-free DMEM and added to intestinal cell monolayers at a concentration of 1 × 10^8^ CFU/well (approximately 100:1 bacteria-to-cell ratio). Co-cultures of bacteria and Caco-2 cells were incubated at 37 °C for 1.5 h. Non-adhering bacteria were removed by 5 washes with Hanks’ Balanced Salt Solution (Corning), and then cell monolayers were lysed with 1% Triton-X-100, according to Schifano et al. [28]. Adhering, viable bacterial cells were quantified by plating appropriate serial dilutions of Caco-2 lysates on MRS agar and incubating for 48 h.

### 2.11. Pathogen Adhesion Assay to Caco-2 Cells in the Presence of L. plantarum Pic37.4 CFS

The pathogen adhesion assay was performed according to Zinno et al. [29], with modifications. Caco-2 cells, seeded and differentiated as described above, were placed in an antibiotic- and FBS-free DMEM 16 h before the assay. On the day of the assay, overnight bacterial cultures of the pathogen indicator strains *L. monocytogenes* OH, *S.* Typhimurium LT2 and ETEC K88 were diluted 1:10 in appropriate media, grown for 2–4 h up to the exponential growth phase, resuspended in antibiotic- and FBS-free DMEM and added to the cell monolayers at a concentration of 1 × 10^7^ CFU/well, alone or in combination with 500 µL of either *L. plantarum* Pic37.4 CFS or CFS (N), corresponding to 1 × 10^9^ CFU/well (approximately 100:1 LAB-to-pathogen ratio). As a control, the same volume of MRS (A) was also used. Co-cultures of bacteria and Caco-2 cells were incubated at 37 °C for 1.5 h. Non-adhering bacteria were then removed and cells lysed as described above. Adhering, viable bacterial cells were quantified by plating appropriate serial dilutions of Caco-2 lysates on different media and incubating for 18 h.

### 2.12. Statistical Analysis

The statistical significance of the differences was evaluated by one-way ANOVA followed by Tukey’s HSD *post hoc* test, after verifying normality and homogeneity of variance by Shapiro–Wilk’s and Levene’s tests, respectively. In case the homogeneity of variance was verified while normality was not, the Kruskal–Wallis test and Dunn’s *post hoc* test were used. In the opposite case (normality verified and homogeneity of variance not verified), Welch one-way ANOVA, followed by Tamhane’s *post hoc* test, was used. For experiments of resistance to *in vitro* digestion, Student’s *t*-test was applied. In the figures, mean values with different superscript letters or asterisks (in ANOVA or *t*-test, respectively) significantly differ (*p* < 0.05). Statistical analyses were executed with Microsoft Office Excel 2011 upgraded with XLSTAT (ver. 4 March 2014). For the assays performed in *C. elegans*, survival analysis was conducted using the Kaplan–Meier method, and differences between groups were assessed with the log-rank (Mantel–Cox) test (GraphPad Prism 9.0 software, GraphPad Software Inc., San Diego, CA, USA). Differences with *p*-values < 0.05 were considered significant and were indicated as follows: * *p* < 0.05, ** *p* < 0.01 and *** *p* < 0.001.

## 3. Results

### 3.1. Isolation, Characterization and Selection of Lactic Acid Bacteria from Pecorino di Picinisco

Starting from a collection of 40 LAB isolated from a sample of Pecorino di Picinisco PDO cheese, morphologically characterized, strain typed through rep-PCR and characterized at the species level (Table S1 and Supplementary Methods), 11 strains displaying unique fingerprinting profiles were identified (Table S2) and associated with the following 3 species: *Lactococcus lactis* (7 strains), *Lactiplantibacillus plantarum* (1 strain) and *Latilactobacillus curvatus* (3 strains). Given the importance of the three species in terms of potential probiotic and technological properties, one representative strain for each of them, namely *L. curvatus* Pic37.1, *Lc. lactis* Pic37.3 and *L. plantarum* Pic37.4, was selected for subsequent analyses, using the well-known commercial LGG as a reference probiotic.

### 3.2. Antibiotic Susceptibility Profile of the Three Selected LAB Strains

The safety profile of the three strains was verified by analyzing susceptibility to the antibiotics recommended by EFSA guidelines [21]. The analysis revealed that all strains tested, including LGG, were susceptible to ampicillin, gentamicin, erythromycin, clindamycin, chloramphenicol and tetracycline, except for *Lc. lactis* Pic37.3, which showed intermediate susceptibility to tetracycline (Table 1). *Lc. lactis* Pic37.3 was the only one tested for vancomycin and resulted susceptible. The strains *L. curvatus* Pic37.1, *L. plantarum* Pic37.4, as well as the LGG strain were presumptively resistant to kanamycin, while *Lc. lactis* Pic37.3 showed susceptibility. On the other hand, *L. curvatus* Pic37.1 and *Lc. lactis* Pic37.3 were presumptively resistant to streptomycin, while LGG showed intermediate susceptibility (Table 1). Overall, the strains were sensitive to most of the antibiotics tested and the antibiotic susceptibility profiles were almost completely overlapping with that of the reference probiotic.

### 3.3. Adhesion Capacity to Human Intestinal Caco-2 Cells of the Three Selected LAB Strains

**Figure 1:**
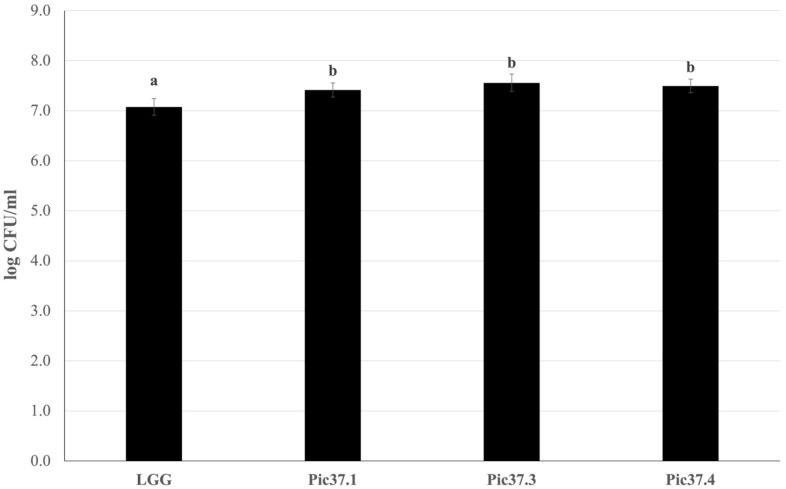
LAB adhesion to Caco-2 cells. Cell counts of viable *Latilactobacillus curvatus* Pic37.1 (Pic37.1), *Lactococcus lactis* Pic37.3 (Pic37.3), *Lactiplantibacillus plantarum* Pic37.4 (Pic37.4) and *Lacticaseibacillus rhamnosus* GG (LGG) adhering to differentiated Caco-2 cells. The initial bacterial load was 1 × 10^8^ colony-forming units (CFU)/well. Data are reported as log CFU/mL recovered after plating. Columns represent the mean ± SD of three independent experiments, each performed at least in duplicate. For statistical analysis, Kruskal–Wallis with Dunn *post hoc* tests were performed (*p* < 0.01). Distinct letters indicate statistically significant differences.

Since adhesion to the intestinal mucosa is one of the necessary requirements for a microorganism to be defined as probiotic, the adhesion capacity of the three LAB strains was evaluated on Caco-2 cells, widely used as a model of human intestinal epithelium [27,30]. The well-known probiotic LGG strain, able to efficiently adhere to Caco-2 cells, was used as a reference to compare the resulting adhesion capacity of the tested LAB strains. Each bacterial strain, namely LGG, *L. curvatus* Pic37.1, *Lc. lactis* Pic37.3 and *L. plantarum* Pic37.4, was added to Caco-2 cells at the same initial concentration of 8 log CFU/mL. The results, shown in Figure 1, are expressed as log CFU/mL recovered at the end of the co-incubation of bacteria with Caco-2 cells. Notably, while adhered LGG was 7.1 log CFU/mL, adhered *L. curvatus* Pic37.1, *Lc. lactis* Pic37.3 and *L. plantarum* Pic37.4 strains were 7.4, 7.6 and 7.5 log CFU/mL, respectively, resulting in significantly higher values than LGG (*p* < 0.01, Figure 1), therefore suggesting promising adhesion properties.

### 3.4. Resistance to Simulated In Vitro Digestion of the Three Selected LAB Strains

**Figure 2:**
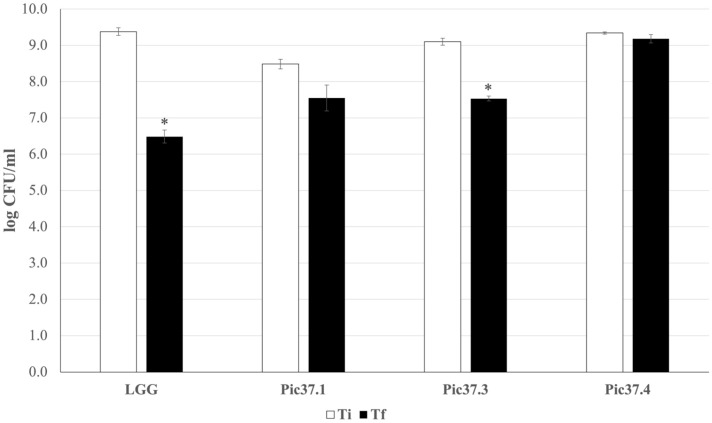
*In vitro* tolerance of LAB to simulated gastrointestinal conditions. Cell counts of viable *Latilactobacillus curvatus* Pic37.1 (Pic37.1), *Lactococcus lactis* Pic37.3 (Pic37.3), *Lactiplantibacillus plantarum* Pic37.4 (Pic37.4) and *Lacticaseibacillus rhamnosus* GG (LGG) at the initial time point (Ti, white columns) and at the end of digestion (Tf, black columns) are shown. Data are reported as log colony-forming units (CFU)/mL recovered after plating bacterial cells at the beginning (initial time point: Ti) and at the end (final time point: Tf) of the simulated digestion process. Columns represent the mean ± SD of two independent experiments, each performed in duplicate. Statistical analysis was performed by Student’s *t*-test (* *p* < 0.05, Tf versus Ti for each strain).

An *in vitro* digestion simulation test was performed to analyze the ability of the three strains to survive the harsh conditions of the gastrointestinal tract, as this capacity represents an important probiotic feature. The results, reported in Figure 2, are expressed as log CFU/mL recovered after plating bacterial cells at the beginning (Ti) and at the end (Tf) of the simulated digestion process for each LAB strain tested and for the probiotic reference LGG. In particular, *L. plantarum* Pic37.4 maintained its concentration almost unchanged throughout the entire digestion process (about 9 log CFU/mL), while LGG, *Lc. lactis* Pic37.3 and *L. curvatus* Pic37.1 showed a reduction of approximately 3, 2.5 and 1 log units, respectively (*p* < 0.05 Tf versus Ti for LGG and *Lc. lactis* Pic37.3, Figure 2). To better compare the results obtained, survival capacity was calculated for each strain and expressed as a percentage. Overall, the capacity of the tested strains to tolerate gastrointestinal conditions ranged between 83 and 98%. Notably, all three strains showed survival capacities higher than that of the reference probiotic strain LGG (70%), with *L. plantarum* Pic37.4 showing the highest value (Table 2).

### 3.5. Evaluation of the Health-Promoting Features of the Three LAB Strains in the Simplified C. elegans In Vivo Model

To evaluate *in vivo* the health-promoting features of the LAB strains, the simplified *C. elegans* model was employed, based on the well-established effectiveness of probiotic bacteria to promote its prolongevity [31,32].

**Figure 3:**
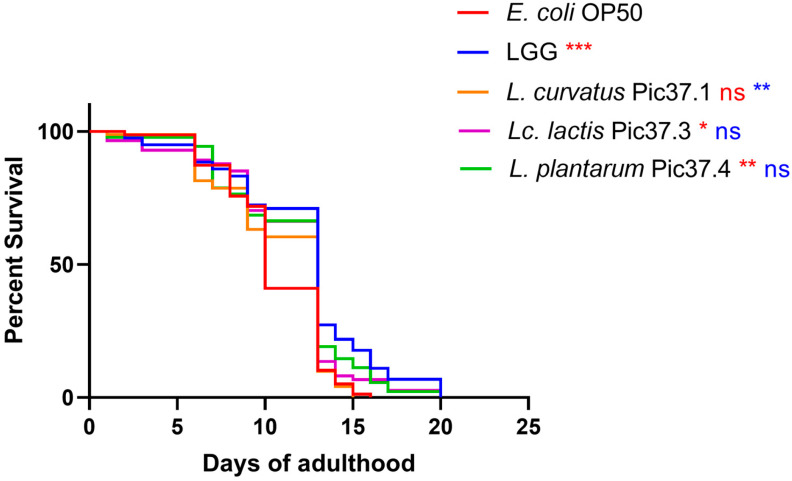
Lifespan of *Caenorhabditis elegans* fed LAB strains from embryo hatching. Kaplan–Meier survival plots of worms fed the three LAB strains and *Escherichia coli* OP50 and *Lacticaseibacillus rhamnosus* GG (LGG), used as controls (*n* = 80 per condition in each experiment). Experiments were conducted in triplicate. Differences between groups were assessed with the log-rank (Mantel–Cox) test. Red and blue asterisks indicate significant differences compared to *E. coli* OP50 and LGG controls, respectively (* *p* < 0.05, ** *p* < 0.01, *** *p* < 0.001); ns: not significant.

To this aim, *C. elegans* lifespan tests were conducted on nematodes separately fed each of the LAB strains starting from embryo hatching by comparing the survival curves with those of worms grown on LGG (probiotic control) or on the standard *E. coli* OP50 diet. The results reported in Figure 3 showed that, among the three LAB strains, *L. plantarum* Pic37.4 and *Lc. lactis* Pic37.3 exerted a significant increase in nematode lifespan as compared to *E. coli* OP50, with a survival curve almost overlapping with that of animals fed LGG (*p* < 0.05 and *p* < 0.01 vs. OP50, respectively). On the other hand, the lifespan of nematodes fed *L. curvatus* Pic37.1 was similar to that observed for animals fed *E. coli* OP50.

Given the excellent ability to survive digestive stress and to extend the nematode lifespan, the *L. plantarum* Pic37.4 strain was therefore selected as the most promising candidate. Therefore, subsequent experiments were carried out on this strain.

### 3.6. Effectiveness of L. plantarum Pic37.4 Against Pathogen and Spoilage Bacteria

Antagonistic activity was evaluated against indicator strains representative of the most common foodborne and intestinal pathogens, namely *L. monocytogenes* OH, *S.* Typhimurium LT2, and ETEC K88, as well as spoilage bacteria, *P. putida* WCS358 and KT2440. Experiments were carried out using intact chloroform-inactivated *L. plantarum* Pic37.4 cells, as well as its CFS, representing the liquid fraction obtained after removing live bacterial cells by centrifugation and filtration following an overnight growth until early stationary phase. Chloroform-inactivated bacteria exert antagonism mainly through structural components, as chloroform disrupts cellular membranes, whereas CFS contains only secreted antimicrobial metabolites by live bacteria.

Chloroform-inactivated *L. plantarum* Pic37.4 was able to inhibit the growth of all indicator strains tested, although with different efficacies (Table 3). *L. monocytogenes* OH and *S.* Typhimurium LT2 exhibited inhibition halos of 5.6 and 5.4 cm in diameter, respectively, indicating a considerable susceptibility to the antimicrobial compound(s) produced by the LAB strain. In the case of ETEC K88, an inhibition diameter of 3.9 cm was observed, while the two spoilage *P. putida* strains showed different responses: the WCS358 strain presented the largest inhibition halo, with a diameter of 9.0 cm, while the KT2440 strain showed a smaller halo of 3.4 cm diameter. To exclude any possible inhibitory effect due to residual chloroform, complete evaporation of chloroform was verified (see Section 2).

A similar experiment was performed with *L. plantarum* Pic37.4 CFS and CFS (N). The results of the inhibition halos showed that CFS exerted an inhibitory effect on all the tested indicator strains, particularly against ETEC K88 (Table S3). In contrast, CFS (N) did not show any inhibition zones in any of the tested strains, suggesting that the inhibitory effect could be attributable to the acidity of the medium, caused by the organic acids produced by *L. plantarum* Pic37.4 metabolism.

**Figure 4:**
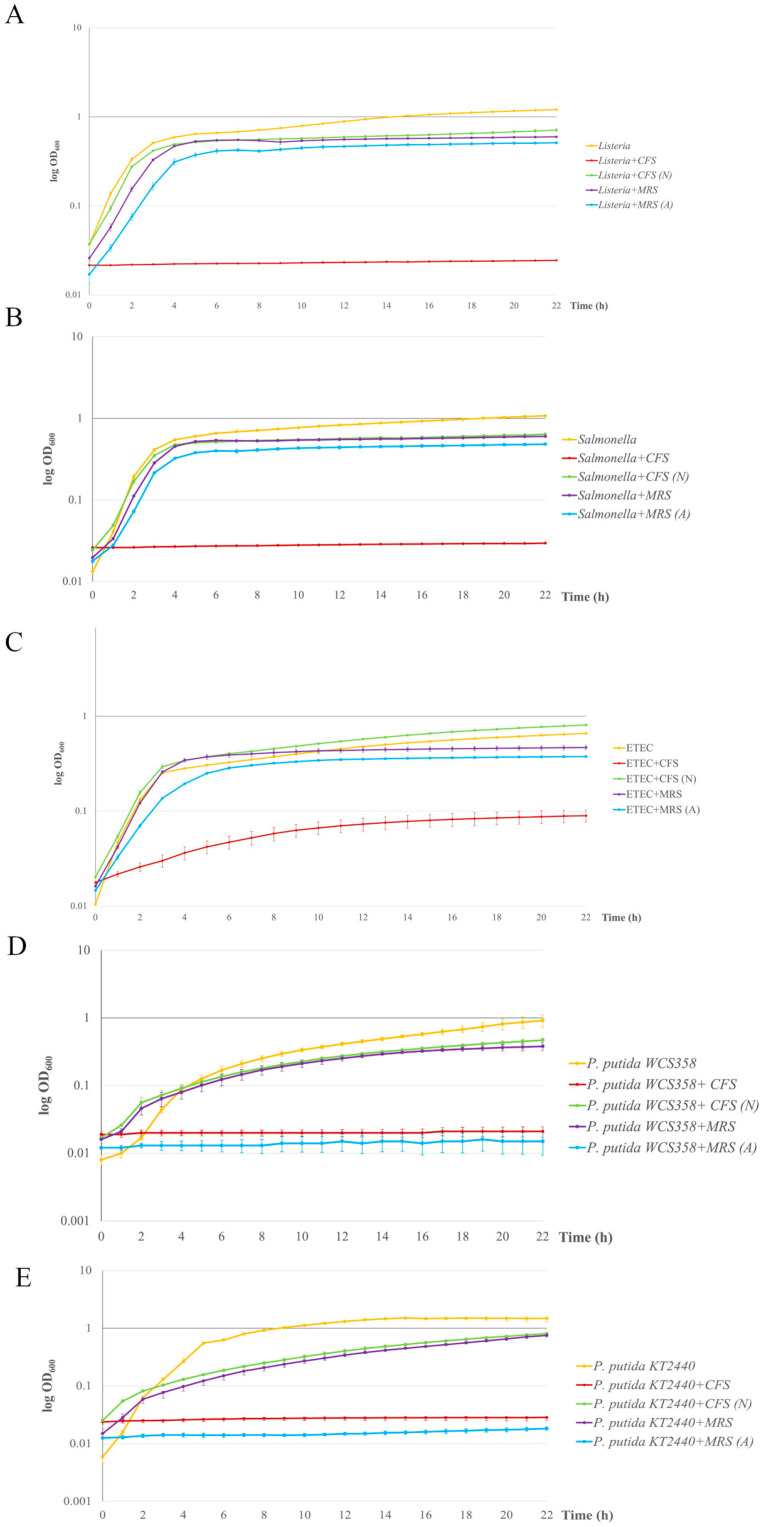
Antimicrobial activity of *Lactiplantibacillus plantarum* Pic37.4 cell-free supernatant. Growth curves of pathogens and spoilage bacteria grown in the presence of *L. plantarum* Pic37.4 cell-free supernatant (CFS, red lines); CFS neutralized to pH 6.5 (CFS (N), green lines); MRS (purple lines); MRS acidified to pH 4 (MRS (A), blue lines). Orange lines refer to reference growth curves of *Listeria monocytogenes* OH (**A**), *Salmonella enterica* serovar Typhimurium LT2 (**B**), enterotoxigenic *Escherichia coli* (ETEC) K88 (**C**), *Pseudomonas putida* WCS358 (**D**) and *Pseudomonas putida* KT2440 (**E**). Bacterial growth was monitored by measuring the OD_600_ at 1 h intervals for 22 h and expressed as log OD_600_. Experiments were performed in triplicate with at least one independent experiment for each strain tested.

To obtain more quantitative results of the antimicrobial activity of *L. plantarum* Pic37.4 CFS, a liquid broth assay was conducted by monitoring the evolution of the growth curves of each indicator strain in the presence of CFS or CFS (N), including as additional controls also MRS medium and MRS adjusted to pH 4 (acidified MRS, MRS (A)), corresponding to the pH of *L. plantarum* Pic37.4 CFS. The assay was performed with 20 or 50 µL of CFS, CFS (N) and relative controls, and the results are shown in Figure 4. In particular, the 20 µL dose of CFS exerted an inhibitory effect exclusively against ETEC K88 (Figure 4C, red line), resulting in a growth curve with a markedly slower trend, while it did not influence the growth of the other indicator strains, confirming the preliminary findings of a dose-dependent effect. Moreover, this result also confirmed that ETEC K88 is particularly vulnerable to one or more bioactive metabolites produced by *L. plantarum* Pic37.4. The addition of 50 µL of CFS determined a strong inhibition of the growth of all the other pathogenic or spoilage strains tested, namely *L. monocytogenes* OH, *S.* Typhimurium LT2, *P. putida* WCS358 and KT2440 (Figure 4A,B,D,E, red lines). As already evidenced in previous experiments, no effect could be observed with CFS (N) for any of the tested indicator strains (Figure 4A–E, green lines). Interestingly, MRS (A) had the same inhibitory effect of CFS on the growth of the two *P. putida* strains (Figure 4D,E, light blue lines), while it did not exert any inhibition on the three pathogen strains (Figure 4A–C, light blue lines), suggesting a particular sensitivity of the *P. putida* strains to acidic environments. Indeed, while the inhibitory effect on the growth of the two spoilage strains could be exclusively ascribable to the acidic pH of CFS and MRS (A), some other factors secreted by *L. plantarum* Pic37.4, active only in acidic environments, could be selectively effective against *L. monocytogenes* OH, *S.* Typhimurium LT2 and ETEC K88, with a greater effect on the latter. Overall, these results suggest that the CFS inhibition involves both organic acids and bioactive components effective in acidic environments, losing efficacy at neutral pH.

### 3.7. Reduction In Pathogen Adhesion to Caco-2 Cells Mediated by L. plantarum Pic37.4 CFS

**Figure 5:**
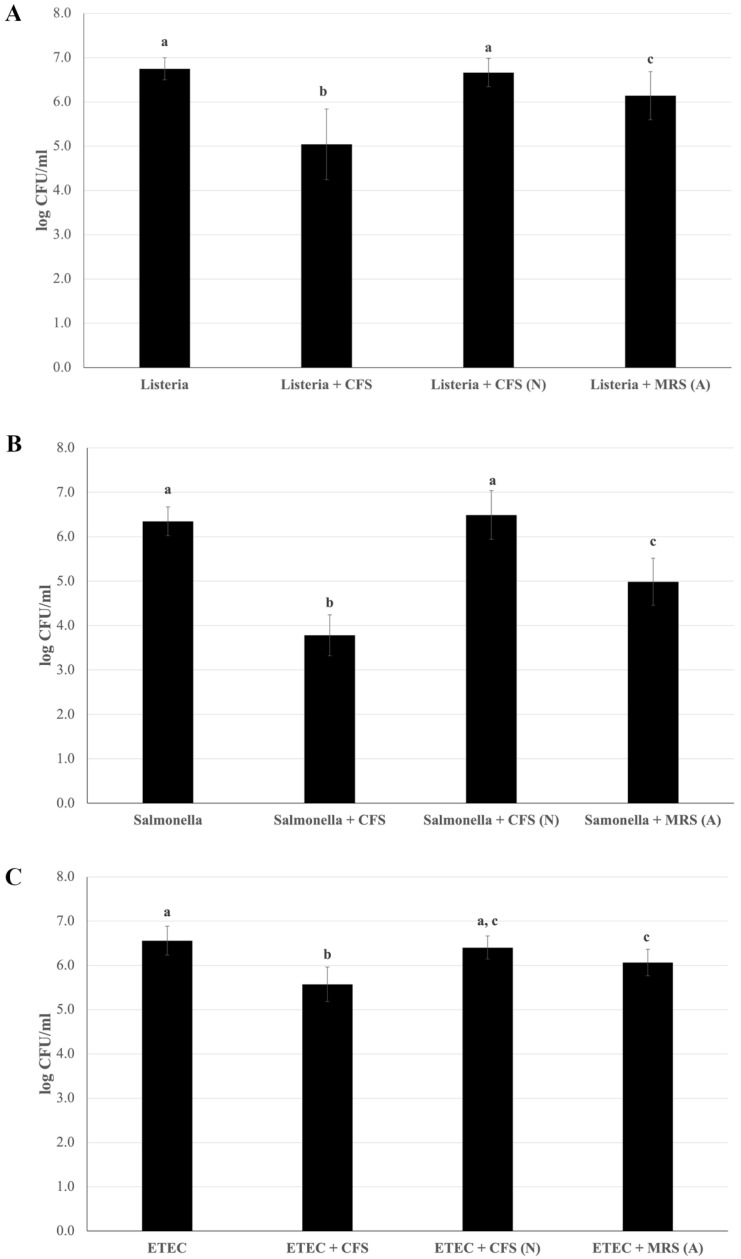
Reduction in pathogen adhesion to Caco-2 cells mediated by *Lactiplantibacillus plantarum* Pic37.4 cell-free supernatant. Viable counts (expressed as means and relative standard deviations of log CFU/mL) of pathogen strains after adhesion to Caco-2 cells alone or in the presence of cell-free supernatant (CFS); CFS neutralized to pH 6.5 (CFS (N)); MRS acidified to pH 4: MRS (A). The pathogens are *Listeria monocytogenes* OH (**A**), *Salmonella enterica* serovar Typhimurium LT2 (**B**) and enterotoxigenic *Escherichia coli* (ETEC) K88 (**C**). Experiments were performed in duplicate, with three independent experiments for each pathogen. Statistical analysis was evaluated by (**A**) Welch one-way ANOVA with the Tamhane *post hoc* test (*p* ≤ 0.001, *Listeria* + CFS versus *Listeria, Listeria* + CFS (N) and *Listeria* + MRS (A); *p* ≤ 0.05, *Listeria* + MRS (A) versus *Listeria* and *Listeria* + CFS (N)); (**B**) one-way ANOVA (*p* < 0.001); and (**C**) one-way ANOVA (*p* < 0.001, ETEC versus ETEC + CFS; *p* < 0.01, ETEC + MRS (A) versus ETEC and ETEC + CFS; ETEC + CFS versus ETEC + CFS (N)). Distinct letters indicate statistically significant differences.

An essential requirement for selecting novel probiotic strains is their ability to counteract the adhesion of pathogens at the intestinal level. To determine whether the LAB strain could release bioactive metabolites into the culture medium able to perform this function, the capacity of *L. plantarum* Pic37.4 CFS in counteracting pathogen adhesion was investigated in Caco-2 cells. All three pathogenic strains were preliminarily tested for their ability to adhere to Caco-2 cells, resulting in comparable adhesion capacities. Figure 5 shows the results of the adhesion inhibition assays, indicating that the presence of *L. plantarum* Pic37.4 CFS significantly reduced the adhesion of the three pathogenic strains analyzed compared to the pathogens alone (*p* < 0.001), with the strongest effect observed for *S.* Typhimurium LT2, whose adhesion was reduced by 2.6 log CFU/mL (Figure 5B). The CFS-induced reduction in adhesion was about 1.7 log CFU/mL for *L. monocytogenes* OH (Figure 5A) and 1 log CFU/mL for ETEC K88 (Figure 5C). On the other hand, the addition of CFS (N) did not reduce the adhesion of the three pathogens (Figure 5A–C), indicating that the CFS lost its remarkable inhibitory activity when neutralized to pH 6.5, highlighting the importance of pH in maintaining its efficacy. Concerning MRS (A), it exerted an inhibitory effect on all three pathogens, compared to the pathogens alone, although to a lesser extent with respect to CFS: indeed, adhesion was reduced by 0.6, 1.4 and 0.5 log CFU/mL for *L. monocytogenes* OH, *S.* Typhimurium LT2 and ETEC K88, respectively, confirming that, in addition to acidity, other factors present in CFS, such as bioactive components secreted by *L. plantarum* Pic37.4, may be involved in counteracting pathogen adhesion.

### 3.8. Evaluation of the Protective Features of the L. plantarum Pic37.4 Against Pathogens in C. elegans

**Figure 6:**
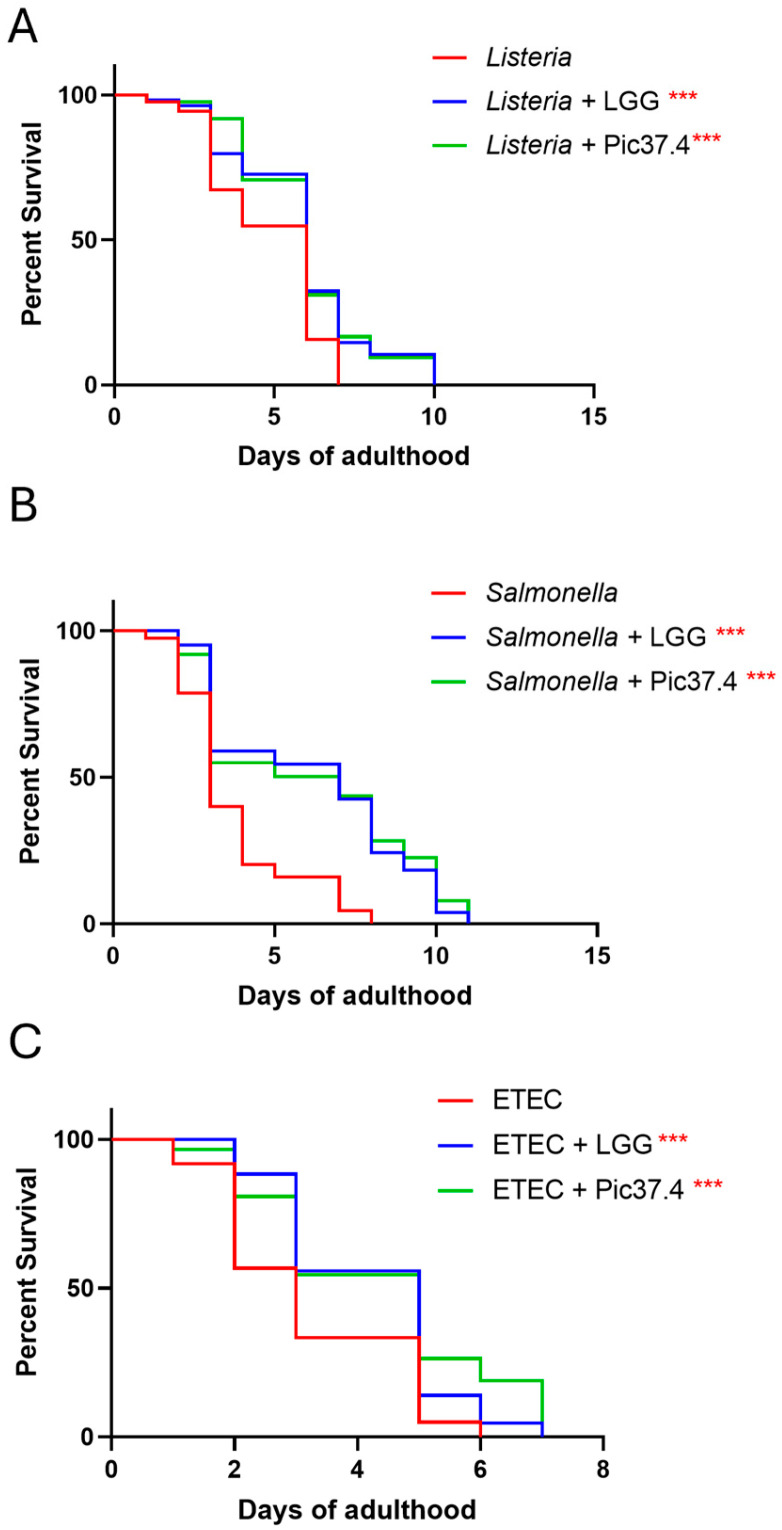
*In vivo* ability of *Lactiplantibacillus plantarum* Pic37.4 to counteract pathogen infection. Kaplan–Meier survival plots of *C. elegans* fed *L. plantarum* Pic37.4 in a 1:1 co-culture with *Listeria monocytogenes* OH (**A**), *Salmonella enterica* serovar Typhimurium LT2 (**B**), or enterotoxigenic *Escherichia coli* (ETEC) K88 (**C**). Experiments were conducted in triplicate. Differences between groups were assessed with the log-rank (Mantel–Cox) test. Worms fed the pathogen alone served as control (*** *p* < 0.001; *n* = 80 per condition in each experiment).

On the basis of the *C. elegans* model as a reliable tool to verify the ability of probiotic bacteria to promote protection against pathogen infection *in vivo* [33], the antagonistic activity of *L. plantarum* Pic37.4 against *L. monocytogenes* OH, *S.* Typhimurium LT2, or ETEC K88 infection was evaluated in nematodes. The results, reported in Figure 6, showed that *L. plantarum* Pic37.4 conferred a protective effect against all the tested pathogens, significantly enhancing worm survival as compared to nematodes exposed to the pathogens alone (*p* < 0.001, Figure 6). Specifically, in the case of *L. monocytogenes* OH, the co-feeding with *L. plantarum* Pic37.4 extended 50% worm survival to day 5, as compared to day 4 of the *L. monocytogenes* OH group (*p* < 0.001, Figure 6A). A significant reduction in *C. elegans* survival was observed after infection with *S.* Typhimurium LT2 alone (50% survival reached at day 2, *p* < 0.001), compared to nematodes exposed to co-cultures of the same pathogen with *L. plantarum* Pic37.4 (50% survival at day 5, *p* < 0.001, Figure 6B). Concerning worms fed ETEC K88 alone, the animals reached 50% survival by day 2, whereas those co-fed with *L. plantarum* Pic37.4 reached the same threshold only by day 3 (*p* < 0.001, Figure 6C). Taken together, these results confirm the *in vivo* ability of *L. plantarum* Pic37.4 to counteract the infection caused by the three pathogens considered in this study.

## 4. Discussion

The aim of the present study was to explore the microbiological properties of Pecorino di Picinisco, an Italian traditional PDO cheese obtained from ovine raw milk, by characterizing its indigenous LAB community and by evaluating the possible probiotic and technological activities of selected strains. Overall, strain typing of the collection of 40 LAB revealed a high level of biodiversity, consistent with findings reported in the literature concerning microbiota of raw milk cheeses [34]. Within the established LAB collection, three strains were selected as representative of the three different species: *L. curvatus* Pic37.1, *Lc. lactis* Pic37.3, and *L. plantarum* Pic37.4. Their safety of use, resistance to simulated *in vitro* gastrointestinal conditions, adhesion ability to intestinal epithelial cells and *C. elegans* lifespan extension were assessed. The overall antibiotic susceptibility profile of the three strains to a panel of antibiotics recommended by EFSA guidelines [21] resulted mostly overlapping with that of the reference probiotic LGG, suggesting their safe use. However, it should be pointed out that, in case of use in the food or pharmaceutical sectors, complete genome sequencing will be essential to exclude the presence of transferable antibiotic resistance genes.

The ability of a bacterial strain to adhere to the intestinal epithelium is crucial to exert beneficial effects in the host intestine, such as competitive exclusion of pathogens and immunomodulation [35], and therefore represents a key probiotic feature. Moreover, epithelial adhesion promotes the persistence of LAB strains in the intestine, enhancing the action of their metabolites, such as SCFAs, which are essential for maintaining intestinal homeostasis [36]. The ability of the three selected LAB strains to adhere to the intestinal epithelium was assessed through a well-established *in vitro* model, the human intestinal Caco-2 cells [27], largely used to evaluate the adhesion capacity of putative probiotic strains [37,38]. The adhesion ability of the three LAB strains to Caco-2 cells resulted in very similar results to each other and even higher than that of the reference probiotic LGG, suggesting promising adhesive properties. The results obtained are consistent with other adhesion experiments on Caco-2 cells reported in the literature concerning *L. curvatus* [39], *Lc. lactis* [36], and *L. plantarum* [40] strains.

To further evaluate the ability of the three LAB strains to reach the intestine in a viable form, their resistance to gastrointestinal stress factors was assessed through a simulated *in vitro* digestion assay. The survival rates of all strains were higher as compared to that of the reference strain LGG, whose survival rate was consistent with previous findings [41]. Overall, the results obtained suggest an excellent capacity of the three tested strains to tolerate adverse conditions of the gastrointestinal tract. In particular, *L. plantarum* Pic37.4 showed the highest survival capacity.

*In vivo* tests using the *C. elegans* model, which represents a powerful tool for high-throughput screening of candidate probiotics [42], also demonstrated that *L. plantarum* Pic37.4 significantly extended the nematode lifespan compared to the other tested LAB strains. This result is consistent with a recent study that screened LAB strains with high antioxidant activity from Tibetan traditional fermented yak milk and investigated their safety and anti-aging effects on oxidative senescence in *C. elegans* [43].

Based on the remarkably promising features demonstrated *in vitro* and *in vivo*, *L. plantarum* Pic37.4 was therefore selected for subsequent experiments, also because the *L. plantarum* species is well-known for including probiotic and bioprotective strains, able to produce antimicrobial molecules, such as bacteriocins, commonly defined as plantaricins [44]. The efficacy of *L. plantarum* Pic37.4 as a bioprotective agent was tested against representative strains of the most common foodborne and intestinal pathogens, as well as spoilage microorganisms. The results showed that both chloroform-inactivated cells and CFS of *L. plantarum* Pic37.4 exhibited remarkable antagonistic activity against the tested pathogens and spoilers, indicating broad-spectrum efficacy against Gram-positive and Gram-negative species. In particular, the CFS of *L. plantarum* Pic37.4, characterized by an acidic pH, strongly inhibited the growth of indicator bacteria, particularly ETEC K88, while the CFS neutralized to pH 6.5 did not show antimicrobial activity. Additionally, MRS medium acidified to pH 4, equivalent to that of the CFS, caused inhibition only of *P. putida* strains’ growth. Such an effect could be explained by the limited tolerance to acidity characteristic of spoilage bacteria, whose growth is typically inhibited at pH levels below 4.5. Indeed, differently from enteric pathogens, spoilers usually live in less extreme environments (soil, water, plant surfaces), resulting in the lack of complex adaptive mechanisms for acid resistance.

Taken together, the results indicate that acidity contributes to the inhibitory effect but is not the unique factor, suggesting the possible presence of bioactive metabolites secreted by *L. plantarum* Pic37.4, active in acidic environments and inactive at neutral pH. Future studies should focus on characterizing such bioactive compounds to better understand the underlying mechanisms that act selectively in some species but not in others. It is also worth noting that, in the case of chloroform-inactivated bacterial cells, some structural components, absent in CFS, could also be implicated in the antagonistic activity, and this could explain the different effect observed against *P. putida* WCS358, which resulted in the most affected strain.

The antimicrobial activity of CFS from different *L. plantarum* strains has been investigated by other authors, with contrasting results, pointing at its effectiveness against other pathogenic and spoilage bacteria [45,46,47] or, on the other hand, proving its inefficacy with respect to intact cells [48]. These findings corroborate the evidence of the strain dependency of probiotic/technological features and support the need for a case-by-case analysis to select strains able to inhibit or displace a specific pathogen [49,50,51].

Experiments assessing pathogen adhesion to Caco-2 intestinal cells in the presence of *L. plantarum* Pic37.4 CFS demonstrated that it significantly reduced pathogen adhesion to the cell monolayer, particularly for *S.* Typhimurium LT2. Acidified MRS medium also showed adhesion inhibition, although less effectively, suggesting that this effect is principally due to acidity, as corroborated by the fact that the neutralized CFS did not induce any effect, indicating that inhibitory activity is lost when the pH approaches neutrality. Given the antimicrobial effect exerted by CFS in liquid broth assay, we can hypothesize that the inhibition of pathogen adhesion to Caco-2 cells could be principally mediated by counteracting pathogen viability, although we cannot exclude that other mechanisms, directed to intestinal cells, could also be involved. These unexplored aspects deserve further investigations. Our findings are in line with previously published results, demonstrating the ability of CFS of *L. plantarum* CS24.2 to significantly reduce *E. coli* O26:H11 adhesion to HT-29 intestinal cells [52]. Moreover, in a recent work, the CFS of *L. plantarum* TW57-4, a probiotic strain isolated from yellow kashk (a popular homemade Persian fermented food), was demonstrated to reduce *L. monocytogenes* adhesion and invasion in HT-29 cells [53]. Assessing the efficacy of inanimate bacterial cells or CFS opens the possibility of using the beneficial strain as a postbiotic, defined as a “preparation of inanimate microorganisms and/or their components that confers a health benefit on the host” [54], allowing us to overcome, for example, the problem of probiotic vitality and stability, as well as the risk of transmitting antibiotic-resistance genes [55].

Results obtained in the *C. elegans* model corroborated the antagonistic activity of *L. plantarum* Pic37.4, demonstrating its effectiveness also *in vivo*, as it significantly conferred protection against pathogen-induced mortality, further supporting its potential probiotic properties. Specifically, *L. plantarum* Pic37.4-fed nematodes exhibited increased resistance to infection caused by *S.* Typhimurium LT2, *L. monocytogenes* OH or ETEC K88. Indeed, *C. elegans* is a valuable model for investigating host-microbe interactions with various human and animal pathogens, including the above-mentioned ones, which can colonize the worm’s gut and establish infection [56,57,58]. The obtained results align with previous studies highlighting the role of LAB in enhancing host resilience against infections by modulating gut microbiota composition and interfering with pathogen colonization (reviewed by Roselli et al. [14]). Moreover, probiotic strains have been shown to act as competitive exclusion agents by adhering to the intestinal mucosa, producing bacteriocins, and creating an acidic environment that inhibits pathogen growth [59].

Taken together, the results of the present work highlight the microbiological properties of Pecorino di Picinisco, focusing on selected LAB strains from its fermenting microbiota that have been characterized in terms of potential probiotic and technological features. Moreover, our findings corroborate the existing knowledge of *L. plantarum* as a versatile species within lactobacilli, resulting in its attractiveness as a promising producer of several antimicrobial compounds that are key in exerting probiotic, strain-specific features [60].

## 5. Conclusions

The three LAB strains isolated from Pecorino di Picinisco cheese have been shown to be promising in terms of probiotic and technological potential, as they displayed good adhesion capacity to human intestinal epithelial Caco-2 cells and were resistant to the harsh conditions of the gastrointestinal tract, laying the ground for further investigations in order to confirm the beneficial characteristics detected. In particular, the *L. plantarum* Pic37.4 strain exhibited the highest survival capacity after digestion, as well as good antimicrobial activities in both *in vitro* and *in vivo* models, indicating interesting technological properties related to its bioprotective activity against major foodborne pathogens and spoilage microorganisms.

Future perspectives include further investigations aimed at identifying and clarifying the nature of the bioactive metabolites secreted by *L. plantarum* Pic37.4 and their interaction with foodborne pathogens. Additionally, validating the effectiveness of this strain in more complex *in vivo* models, such as mice, as well as in clinical trials, will allow for a more in-depth assessment of its health-promoting potential.

## Figures and Tables

**Table 1:** Antibiotic susceptibility profile of *L. curvatus* Pic37.1, *Lc. lactis* Pic37.3 and *L. plantarum* Pic37.4 strains.

Antibiotic	Amount on Disk (μg)	LGG **	*L. curvatus* Pic37.1	*Lc. lactis* Pic37.3	*L. plantarum* Pic37.4
Ampicillin	10	S	S	S	S
Vancomycin	30	N.R.	N.R.	S	N.R.
Gentamicin	30	S	S	S	S
Kanamycin	64	R	R	S	R
Streptomycin	32	I	-	R	N.R.
Streptomycin *	64	-	R	-	-
Erythromycin	15	S	S	S	S
Clindamycin	10	S	S	S	S
Tetracycline	30	S	S	I	S
Chloramphenicol	30	S	S	S	S

* According to EFSA guidelines, the microbiological breakpoint of Streptomycin for *L. curvatus* is 64 μg/mL. ** *L. rhamnosus* GG (LGG) was used as a control strain. For each antibiotic, indication of susceptibility (S), intermediate (I) or resistance (R) is reported, as described in Materials and Methods. “N.R.” indicates “Not Requested” (as the species has intrinsic resistance).

**Table 2:** Survival capacity to *in vitro* gastrointestinal conditions of *L. curvatus* Pic37.1, *Lc. lactis* Pic37.3 and *L. plantarum* Pic37.4 strains.

Bacterial Strain	Survival Capacity
*L. curvatus* Pic37.1	89%
*Lc. lactis* Pic37.3	83%
*L. plantarum* Pic37.4	98%
LGG *	70%

For each strain, survival capacity was calculated as the percentage of 1 − [(log CFU/mL Ti − log CFU/mL Tf)/log CFU/mL Ti]; * L. rhamnosus GG (LGG) was used as the control strain.

**Table 3:** Inhibitory activity of chloroform inactivated *L. plantarum* Pic37.4 against pathogen and spoilage bacteria.

Indicator Strain	Inhibitory Halo Diameter (cm)
*L. monocytogenes* OH	5.6 ± 0
*S*. Typhimurium LT2	5.4 ± 0.2
ETEC K88	3.9 ± 0.1
*P. putida* WCS358	9.0 ± 0
*P. putida* KT2440	3.4 ± 0.3

For each indicator strain tested, the diameters of the halos are expressed in cm and refer to mean ± SD of two independent experiments conducted in duplicate.

## Data Availability

Nucleotide sequences of the amplified 16S rDNA from *Latilactobacillus curvatus* Pic37.1, *Lactococcus lactis* Pic37.3 and *Lactiplantibacillus plantarum* Pic37.4 were deposited in GenBank [https://www.ncbi.nlm.nih.gov/nucleotide/] under accession numbers PV291968, PV291969 and PV291970, respectively.

## Supplementary Materials

The following supporting information can be downloaded at https://www.mdpi.com/article/10.3390/microorganisms13061368/s1, Supplementary Methods; Table S1: List of the 40 isolates from Pecorino di Picinisco; Table S2: List of the 11 representative strains of the collection; Table S3: Inhibitory activity of *L. plantarum* Pic37.4 cell-free supernatant against pathogen and spoilage bacteria.

## Abbreviations

The following abbreviations are used in this manuscript:

CFS	cell-free supernatant
CFS (N)	neutralized cell-free supernatant
CFU	colony-forming units
DMEM	Dulbecco’s Modified Eagle Medium
ETEC	enterotoxigenic *Escherichia coli*
FBS	fetal bovine serum
LAB	lactic acid bacteria
LGG	*Lacticaseibacillus rhamnosus* GG
MRS	De Man Rogosa Sharp
MRS (A)	acidified MRS
PDO	Protected Designation of Origin
*S.* Typhimurium	*Salmonella enterica* serovar Typhimurium

## References

[1] Marco M.L., Heeney D., Binda S., Cifelli C.J., Cotter P.D., Foligné B., Gänzle M., Kort R., Pasin G., Pihlanto A. (2017). Health benefits of fermented foods: Microbiota and beyond. Curr. Opin. Biotechnol..

[2] Mukherjee A., Breselge S., Dimidi E., Marco M.L., Cotter P.D. (2024). Fermented foods and gastrointestinal health: Underlying mechanisms. Nat. Rev. Gastroenterol. Hepatol..

[3] Roselli M., Natella F., Zinno P., Guantario B., Canali R., Schifano E., De Angelis M., Nikoloudaki O., Gobbetti M., Perozzi G. (2021). Colonization Ability and Impact on Human Gut Microbiota of Foodborne Microbes from Traditional or Probiotic-Added Fermented Foods: A Systematic Review. Front. Nutr..

[4] Marco M.L., Sanders M.E., Gänzle M., Arrieta M.C., Cotter P.D., De Vuyst L., Hill C., Holzapfel W., Lebeer S., Merenstein D. (2021). The International Scientific Association for Probiotics and Prebiotics (ISAPP) consensus statement on fermented foods. Nat. Rev. Gastroenterol. Hepatol..

[5] Şanlier N., Gökcen B.B., Sezgin A.C. (2019). Health benefits of fermented foods. Crit. Rev. Food Sci. Nutr..

[6] Carlino N., Blanco-Míguez A., Punčochář M., Mengoni C., Pinto F., Tatti A., Manghi P., Armanini F., Avagliano M., Barcenilla C. (2024). Unexplored microbial diversity from 2,500 food metagenomes and links with the human microbiome. Cell.

[7] Posheva V., Muleshkova T., Josifovska S., Chakarov S., Dimov S.G. (2024). Review on the NGS-based studies of microbiotas of artisanal and regional kinds of cheese with potential as functional foods: Composition and functional analysis. Biotechnol. Biotechnol. Equip..

[8] Tamang J.P., Cotter P.D., Endo A., Han N.S., Kort R., Liu S.Q., Mayo B., Westerik N., Hutkins R. (2020). Fermented foods in a global age: East meets West. Compr. Rev. Food Sci. Food Saf..

[9] Yeluri Jonnala B.R., McSweeney P.L.H., Sheehan J.J., Cotter P.D. (2018). Sequencing of the Cheese Microbiome and Its Relevance to Industry. Front. Microbiol..

[10] Leeuwendaal N.K., Stanton C., O’Toole P.W., Beresford T.P. (2022). Fermented Foods, Health and the Gut Microbiome. Nutrients.

[11] Saxelin M., Lassig A., Karjalainen H., Tynkkynen S., Surakka A., Vapaatalo H., Järvenpää S., Korpela R., Mutanen M., Hatakka K. (2010). Persistence of probiotic strains in the gastrointestinal tract when administered as capsules, yoghurt, or cheese. Int. J. Food Microbiol..

[12] Wang J., Xu Q., Lu C., Cao J., Zhuang L., Li Y., Li Z., Song Y., Zhou S., Zhong F. (2025). Probiotics isolated from the fermented grains of Chinese baijiu alleviate alcohol-induced liver injury by regulating alcohol metabolism and the gut microbiota in mice. Food Funct..

[13] Xiong M.-J., Cui R., Hu T.-G., Wu H. (2025). Hypoglycemic effects of Lactiplantibacillus plantarum B19 via promoting AMPK/PI3K/AKT signaling pathway. Food Biosci..

[14] Roselli M., Schifano E., Guantario B., Zinno P., Uccelletti D., Devirgiliis C. (2019). Caenorhabditis Elegans and Probiotics Interactions from a Prolongevity Perspective. Int. J. Mol. Sci..

[15] Anumudu C.K., Miri T., Onyeaka H. (2024). Multifunctional Applications of Lactic Acid Bacteria: Enhancing Safety, Quality, and Nutritional Value in Foods and Fermented Beverages. Foods.

[16] Sharma H., Ozogul F., Bartkiene E., Rocha J.M. (2023). Impact of lactic acid bacteria and their metabolites on the techno-functional properties and health benefits of fermented dairy products. Crit. Rev. Food Sci. Nutr..

[17] Ibrahim S.A., Ayivi R.D., Zimmerman T., Siddiqui S.A., Altemimi A.B., Fidan H., Esatbeyoglu T., Bakhshayesh R.V. (2021). Lactic Acid Bacteria as Antimicrobial Agents: Food Safety and Microbial Food Spoilage Prevention. Foods.

[18] Castellano P., Melian C., Burgos C., Vignolo G. (2023). Bioprotective cultures and bacteriocins as food preservatives. Advances in Food and Nutrition Research.

[19] Zapaśnik A., Sokołowska B., Bryła M. (2022). Role of Lactic Acid Bacteria in Food Preservation and Safety. Foods.

[20] Rezac S., Kok C.R., Heermann M., Hutkins R. (2018). Fermented Foods as a Dietary Source of Live Organisms. Front. Microbiol..

[21] Rychen G., Aquilina G., Azimonti G., Bampidis V., Bastos M.d.L., Bories G., Chesson A., Cocconcelli P.S., Flachowsky G., EFSA Panel on Additives and Products or Substances used in Animal Feed (FEEDAP) (2018). Guidance on the characterisation of microorganisms used as feed additives or as production organisms. EFSA J..

[22] Vizoso Pinto M.G., Franz C.M.A.P., Schillinger U., Holzapfel W.H. (2006). Lactobacillus spp. with in vitro probiotic properties from human faeces and traditional fermented products. Int. J. Food Microbiol..

[23] Stiernagle T. (2006). Maintenance of C. elegans.

[24] Pompa L., Montanari A., Tomassini A., Bianchi M.M., Aureli W., Miccheli A., Uccelletti D., Schifano E. (2023). In Vitro Probiotic Properties and In Vivo Anti-Ageing Effects of Lactoplantibacillus plantarum PFA2018AU Strain Isolated from Carrots on Caenorhabditis elegans. Microorganisms.

[25] Damaceno Q.S., Souza J.P., Nicoli J.R., Paula R.L., Assis G.B., Figueiredo H.C., Azevedo V., Martins F.S. (2017). Evaluation of Potential Probiotics Isolated from Human Milk and Colostrum. Probiotics Antimicrob. Proteins.

[26] Natoli M., Leoni B.D., D’Agnano I., D’Onofrio M., Brandi R., Arisi I., Zucco F., Felsani A. (2011). Cell growing density affects the structural and functional properties of Caco-2 differentiated monolayer. J. Cell. Physiol..

[27] Sambuy Y., Angelis I.D., Ranaldi G., Scarino M.L., Stammati A., Zucco F. (2005). The Caco-2 cell line as a model of the intestinal barrier: Influence of cell and culture-related factors on Caco-2 cell functional characteristics. Cell Biol. Toxicol..

[28] Schifano E., Zinno P., Guantario B., Roselli M., Marcoccia S., Devirgiliis C., Uccelletti D. (2019). The Foodborne Strain Lactobacillus fermentum MBC2 Triggers pept-1-Dependent Pro-Longevity Effects in *Caenorhabditis elegans*. Microorganisms.

[29] Zinno P., Guantario B., Lombardi G., Ranaldi G., Finamore A., Allegra S., Mammano M.M., Fascella G., Raffo A., Roselli M. (2023). Chemical Composition and Biological Activities of Essential Oils from *Origanum vulgare* Genotypes Belonging to the Carvacrol and Thymol Chemotypes. Plants.

[30] Sambuy Y., Ferruzza S., Ranaldi G., De Angelis I. (2001). Intestinal Cell Culture Models: Applications in Toxicology and Pharmacology. Cell Biol. Toxicol..

[31] Park M.R., Ryu S., Maburutse B.E., Oh N.S., Kim S.H., Oh S., Jeong S.-Y., Jeong D.-Y., Oh S., Kim Y. (2018). Probiotic *Lactobacillus fermentum* strain JDFM216 stimulates the longevity and immune response of *Caenorhabditis elegans* through a nuclear hormone receptor. Sci. Rep..

[32] Yun B., Ryu S., Kang M., Lee J., Yoo J., Kim Y., Oh S. (2022). Probiotic Lacticaseibacillus rhamnosus GG Increased Longevity and Resistance Against Foodborne Pathogens in *Caenorhabditis elegans* by Regulating MicroRNA miR-34. Front. Cell. Infect. Microbiol..

[33] Neuhaus K., Lamparter M.C., Zölch B., Landstorfer R., Simon S., Spanier B., Ehrmann M.A., Vogel R.F. (2017). Probiotic *Enterococcus faecalis* Symbioflor^®^ down regulates virulence genes of EHEC in vitro and decrease pathogenicity in a *Caenorhabditis elegans* model. Arch. Microbiol..

[34] Coelho M.C., Malcata F.X., Silva C.C.G. (2022). Lactic Acid Bacteria in Raw-Milk Cheeses: From Starter Cultures to Probiotic Functions. Foods.

[35] Sylvere N., Mustopa A.Z., Budiarti S., Meilina L., Hertati A., Handayani I. (2023). Whole-genome sequence analysis and probiotic characteristics of *Lactococcus lactis* Subsp. lactis strain Lac3 isolated from traditional fermented buffalo milk (Dadih). J. Genet. Eng. Biotechnol..

[36] Sałański P., Kowalczyk M., Bardowski J.K., Szczepankowska A.K. (2022). Health-Promoting Nature of Lactococcus lactis IBB109 and Lactococcus lactis IBB417 Strains Exhibiting Proliferation Inhibition and Stimulation of Interleukin-18 Expression in Colorectal Cancer Cells. Front. Microbiol..

[37] Alp D., Kuleaşan H. (2019). Adhesion mechanisms of lactic acid bacteria: Conventional and novel approaches for testing. World J. Microbiol. Biotechnol..

[38] Vasiee A., Falah F., Behbahani B.A., Tabatabaee-yazdi F. (2020). Probiotic characterization of Pediococcus strains isolated from Iranian cereal-dairy fermented product: Interaction with pathogenic bacteria and the enteric cell line Caco-2. J. Biosci. Bioeng..

[39] Zommiti M., Connil N., Hamida J.B., Ferchichi M. (2017). Probiotic Characteristics of Lactobacillus curvatus DN317, a Strain Isolated from Chicken Ceca. Probiotics Antimicrob. Proteins.

[40] Sharma S., Kanwar S.S. (2017). Adherence potential of indigenous lactic acid bacterial isolates obtained from fermented foods of Western Himalayas to intestinal epithelial Caco-2 and HT-29 cell lines. J. Food Sci. Technol..

[41] Guantario B., Zinno P., Schifano E., Roselli M., Perozzi G., Palleschi C., Uccelletti D., Devirgiliis C. (2018). In Vitro and in Vivo Selection of Potentially *Probiotic lactobacilli* from Nocellara del Belice Table Olives. Front. Microbiol..

[42] Poupet C., Chassard C., Nivoliez A., Bornes S. (2020). *Caenorhabditis elegans*, a Host to Investigate the Probiotic Properties of Beneficial Microorganisms. Front. Nutr..

[43] Li W., Gao L., Huang W., Ma Y., Muhammad I., Hanif A., Ding Z., Guo X. (2022). Antioxidant properties of lactic acid bacteria isolated from traditional fermented yak milk and their probiotic effects on the oxidative senescence of *Caenorhabditis elegans*. Food Funct..

[44] Fischer S.W., Titgemeyer F. (2023). Protective Cultures in Food Products: From Science to Market. Foods.

[45] Abou Elez R.M.M., Elsohaby I., Al-Mohammadi A.-R., Seliem M., Tahoun A.B.M.B., Abousaty A.I., Algendy R.M., Mohamed E.A.A., El-Gazzar N. (2023). Antibacterial and anti-biofilm activities of probiotic Lactobacillus plantarum against Listeria monocytogenes isolated from milk, chicken and pregnant women. Front. Microbiol..

[46] Wang D., Liu Y., Li X., Chen S., Deng J., Li C., Pan C., Wang Y., Xiang H., Feng Y. (2023). Unraveling the antibacterial mechanism of *Lactiplantibacillus plantarum* MY2 cell-free supernatants against *Aeromonas hydrophila* ST3 and potential application in raw tuna. Food Control.

[47] Wang J., Su Y., Gu L., Chang C., Xu L., Yang Y., Li J. (2021). The inhibition of cell-free supernatants of several lactic acid bacteria on the selected psychrophilic spoilage bacteria in liquid whole egg. Food Control.

[48] Poimenidou S.V., Skarveli A., Saxami G., Mitsou E.K., Kotsou M., Kyriacou A. (2023). Inhibition of Listeria monocytogenes Growth, Adherence and Invasion in Caco-2 Cells by Potential Probiotic Lactic Acid Bacteria Isolated from Fecal Samples of Healthy Neonates. Microorganisms.

[49] Collado M.C., Meriluoto J., Salminen S. (2007). Role of commercial probiotic strains against human pathogen adhesion to intestinal mucus. Lett. Appl. Microbiol..

[50] Fontana L., Bermudez-Brito M., Plaza-Diaz J., Muñoz-Quezada S., Gil A. (2013). Sources, isolation, characterisation and evaluation of probiotics. Br. J. Nutr..

[51] Hill C., Guarner F., Reid G., Gibson G.R., Merenstein D.J., Pot B., Morelli L., Canani R.B., Flint H.J., Salminen S. (2014). The International Scientific Association for Probiotics and Prebiotics consensus statement on the scope and appropriate use of the term probiotic. Nat. Rev. Gastroenterol. Hepatol..

[52] Dhanani A.S., Bagchi T. (2013). *Lactobacillus plantarum* CS24.2 prevents *Escherichia coli* adhesion to HT-29 cells and also down-regulates enteropathogen-induced tumor necrosis factor-α and interleukin-8 expression. Microbiol. Immunol..

[53] Rouhi A., Falah F., Azghandi M., Alizadeh Behbahani B., Mortazavi S.A., Tabatabaei-Yazdi F., Vasiee A. (2024). Investigating the effect of *Lactiplantibacillus plantarum* TW57-4 in preventing biofilm formation and expression of virulence genes in *Listeria monocytogenes* ATCC 19115. LWT.

[54] Salminen S., Collado M.C., Endo A., Hill C., Lebeer S., Quigley E.M.M., Sanders M.E., Shamir R., Swann J.R., Szajewska H. (2021). The International Scientific Association of Probiotics and Prebiotics (ISAPP) consensus statement on the definition and scope of postbiotics. Nat. Rev. Gastroenterol. Hepatol..

[55] Moradi M., Kousheh S.A., Almasi H., Alizadeh A., Guimarães J.T., Yılmaz N., Lotfi A. (2020). Postbiotics produced by lactic acid bacteria: The next frontier in food safety. Compr. Rev. Food Sci. Food Saf..

[56] Aljasir S.F., D’Amico D.J. (2021). Probiotic potential of commercial dairy-associated protective cultures: In vitro and in vivo protection against Listeria monocytogenes infection. Food Res. Int..

[57] Burkhardt W., Salzinger C., Fischer J., Malorny B., Fischer M., Szabo I. (2023). The nematode worm *Caenorhabditis elegans* as an animal experiment replacement for assessing the virulence of different *Salmonella enterica* strains. Front. Microbiol..

[58] Tan K., Deng D., Ma X., Cui Y., Tian Z. (2020). *Pediococcus acidilactici* P25 Protected *Caenorhabditis elegans* against Enterotoxigenic *Escherichia coli* K88 Infection and Transcriptomic Analysis of Its Potential Mechanisms. BioMed Res. Int..

[59] Sharma K., Pooranachithra M., Balamurugan K., Goel G. (2019). Probiotic mediated colonization resistance against *E. coli* infection in experimentally challenged *Caenorhabditis elegans*. Microb. Pathog..

[60] Rocchetti M.T., Russo P., Capozzi V., Drider D., Spano G., Fiocco D. (2021). Bioprospecting Antimicrobials from *Lactiplantibacillus plantarum*: Key Factors Underlying Its Probiotic Action. Int. J. Mol. Sci..

