# Supplementary material for: Isolation and Characterization of Lactic Acid Bacteria from an Italian Traditional Raw Milk Cheese: Probiotic Properties and Technological Performance of Selected Strains
Source: Microorganisms. 2025 Jun 12;13(6):1368. doi: 10.3390/microorganisms13061368 (PMC12196492; doi:10.3390/microorganisms13061368)
Supplement: Supplementary file 1 [file microorganisms-13-01368-s001.zip › Supplementary Methods.pdf]

S 2.1 LAB isolation from cheese

Picinisco cheese samples were purchased from a local supermarket and 10 g were homogenized with 90 ml of 0.9% NaCl for approximately 1 min using a BagMixer400 (Interscience, France). Subsequently, serial dilutions were plated and incubated as follows: presumptive lactobacilli on MRS medium at 30 and 37 °C for 48 h under anaerobic conditions; presumptive cocci on M17 lactose medium at 37 °C for 48 h under anaerobic conditions. A total of 40 colonies were randomly picked and purified by streaking on the corresponding isolation medium. All the isolates were tested for the morphology (bacilli or cocci), Gram reaction and catalase activity. Gram-positive, catalase-negative, bacteria were included in the collection and stored at −80 °C.

S 2.2 Strain and species identification

Repetitive extragenic palindromic (Rep)-PCR with GTG5 primer (5′-GTGGTGGTGGTGGTG-3′) was performed on all bacterial isolates, and the corresponding fingerprintings were analyzed by gel electrophoresis, as previously described (Guantario et al., 2018). For taxonomical identification, 16S rDNA gene fragments were amplified from selected LAB isolates using the P0-P6 primer pair (as described in Guantario et al., 2018). Sequencing of 16S rDNA fragments was performed at Eurofins (Italy) laboratories. For taxonomical identification, DNA sequences were compared with those reported in the BLAST NCBI database. Nucleotide sequences of the amplified 16S rDNA from the three selected LAB strains *Latilactobacillus curvatus* Pic37.1, *Lactococcus lactis* Pic37.3 and *Lactiplantibacillus plantarum* Pic37.4 were submitted to GenBank, and the corresponding accession numbers are PV291968, PV291969 and PV291970, respectively.

S 2.3 In vitro antimicrobial activity of L. plantarum Pic37.4 cell free supernatant (CFS) against pathogen and spoilage bacterial strains by well diffusion assay

For well diffusion assay, the different indicator strains were inoculated at 0.3% (percentage set up in preliminary experiments) in the appropriate soft agar medium. Then, 0.6 cm diameter holes were made in the plates by piercing the soft agar with an agar punch. Eighty µl of *L. plantarum* Pic37.4 CFS or CFS (N) were dispensed into each well. Once the supernatant was absorbed, the plates were incubated for 18 h, and inhibition halos diameters were measured in cm. The well diameter, corresponding to 0.6 cm, was subtracted from the measures.
