# Supplementary material for: Isolation and Characterization of Lactic Acid Bacteria from an Italian Traditional Raw Milk Cheese: Probiotic Properties and Technological Performance of Selected Strains
Source: Microorganisms. 2025 Jun 12;13(6):1368. doi: 10.3390/microorganisms13061368 (PMC12196492; doi:10.3390/microorganisms13061368)
Supplement: Supplementary file 1 [file microorganisms-13-01368-s001.zip › TableS1.pdf]

Table S1. List of the 40 isolates from Pecorino di Picinisco**.** For each isolate, the identification code (ID), the isolation medium and temperature, the observed morphology, and the rep-PCR fingerprinting profiles are indicated. The results of the amplification and sequencing of the 16S rDNA gene for the selected isolates are also provided.

ID	Isolation medium	Isolation temperature (°C)	Morphology	Rep-PCR fingerprinting profile	16S rDNA gene amplification	16S rDNA gene sequencing	Notes
Pic37.1	MRS	37	Bacillary	a	satisfactory	satisfactory	
Pic37.2	MRS	37	Bacillary	b	satisfactory	satisfactory	
Pic37.3	MRS	37	Coccoid	A	satisfactory	satisfactory	
Pic37.4	MRS	37	Bacillary	c	satisfactory	satisfactory	
Pic37.5	MRS	37	Coccoid	E	not performed	not performed	
Pic37.6	MRS	37	Coccoid	F1	satisfactory	unsatisfactory	Excluded
Pic37.7	MRS	37	Bacillary	negative	not performed	not performed	Excluded
Pic37.8	MRS	37	Coccoid	C	not performed	not performed	
Pic37.9	MRS	37	Bacillary	d	not performed	not performed	
Pic37.10	MRS	37	Bacillary	c	negative	not performed	Excluded
Pic37.11	MRS	37	Coccoid	C	satisfactory	negative	Excluded
Pic37.12	MRS	37	Coccoid	A1	negative	not performed	Excluded
Pic37.13	LM17	37	Coccoid	F	satisfactory	satisfactory	
Pic37.14	LM17	37	Coccoid	A2	satisfactory	satisfactory	
Pic37.15	LM17	37	Coccoid	B	satisfactory	satisfactory	
Pic37.16	LM17	37	Coccoid	A	not performed	not performed	
Pic37.17	LM17	37	Coccoid	A1	satisfactory	satisfactory	
Pic37.18	LM17	37	Coccoid	A	not performed	not performed	
Pic37.19	LM17	37	Coccoid	E	satisfactory	satisfactory	
Pic37.20	LM17	37	Coccoid	A	not performed	not performed	
Pic37.21	LM17	37	Coccoid	D	satisfactory	satisfactory	
Pic37.22	LM17	37	Coccoid	A	not performed	not performed	
Pic37.23	LM17	37	Coccoid	unsatisfactory	not performed	not performed	Excluded
Pic37.24	LM17	37	Coccoid	A	not performed	not performed	
Pic30.1	MRS	30	Coccoid	A	not performed	not performed	
Pic30.2	MRS	30	Coccoid	E	not performed	not performed	
Pic30.3	MRS	30	Bacillary	negative	not performed	not performed	Excluded
Pic30.4	MRS	30	Bacillary	negative	negative	not performed	Excluded
Pic30.5	MRS	30	Coccoid	A	not performed	not performed	
Pic30.6	MRS	30	Coccoid	E	not performed	not performed	
Pic30.7	MRS	30	Bacillary	e	negative	not performed	Excluded
Pic30.8	MRS	30	Coccoid	negative	not performed	not performed	Excluded
Pic30.9	MRS	30	Coccoid	A	not performed	not performed	
Pic30.10	MRS	30	Coccoid	E	not performed	not performed	
Pic30.11	MRS	30	Bacillary	d	satisfactory	satisfactory	
Pic30.12	MRS	30	Coccoid	A	not performed	not performed	
Pic30.13	MRS	30	Coccoid	unsatisfactory	not performed	not performed	Excluded
Pic30.14	MRS	30	Coccoid	unsatisfactory	not performed	not performed	Excluded

Pic30.15	MRS	30	Bacillary	f	negative	not performed	Excluded
Pic30.16	MRS	30	Coccoid	A	not performed	not performed	
