# Supplementary material for: Isolation and Characterization of Lactic Acid Bacteria from an Italian Traditional Raw Milk Cheese: Probiotic Properties and Technological Performance of Selected Strains
Source: Microorganisms. 2025 Jun 12;13(6):1368. doi: 10.3390/microorganisms13061368 (PMC12196492; doi:10.3390/microorganisms13061368)
Supplement: Supplementary file 1 [file microorganisms-13-01368-s001.zip › TableS2.pdf]

Table S2. List of the 11 representative strains of the collection. Each strain is indicated by the identification code (ID), species, and rep-PCR fingerprinting profile. The number of isolates within the collection belonging to each rep-PCR fingerprinting profile is reported. The 3 strains selected for experiments on probiotic and technologic characteristics are highlighted in bold.

ID	Species	Rep-PCR fingerprinting profile	Isolates within collection (n)
**Pic37.1**	***Latilactobacillus curvatus***	a	1
Pic37.2	*Latilactobacillus curvatus*	b	1
Pic30.11	*Latilactobacillus curvatus*	d	2
**Pic37.4**	***Lactiplantibacillus plantarum***	c	1
**Pic37.3**	***Lactococcus lactis***	A	11
Pic37.17	*Lactococcus lactis*	A1	1
Pic37.14	*Lactococcus lactis*	A2	1
Pic37.15	*Lactococcus lactis*	B	2
Pic37.21	*Lactococcus lactis*	D	1
Pic37.19	*Lactococcus lactis*	E	5
Pic37.13	*Lactococcus lactis*	F	1
