# Supplementary material for: Isolation and Characterization of Lactic Acid Bacteria from an Italian Traditional Raw Milk Cheese: Probiotic Properties and Technological Performance of Selected Strains
Source: Microorganisms. 2025 Jun 12;13(6):1368. doi: 10.3390/microorganisms13061368 (PMC12196492; doi:10.3390/microorganisms13061368)
Supplement: Supplementary file 1 [file microorganisms-13-01368-s001.zip › TableS3.pdf]

Table S3. Inhibitory activity of *L. plantarum* Pic37.4 cell free supernatant against pathogen and spoilage bacteria.

	***L. plantarum* Pic37.4**	
	CFS	CFS (N)
***L. monocytogenes* OH**	0.46±0.05	0
***S.* Typhimurium LT2**	0.44±0.05	0
**ETEC K88**	0.74±0.15	0
***P. putida* WCS358**	0.44±0.05	0
***P. putida* KT2440**	0.45±0.09	0

CFS: cell free supernatant of *L. plantarum* Pic37.4

CFS (N): CFS neutralized to pH 6.5

For each indicator strain tested, the diameters of the halos are expressed in cm and refer to mean±SD of at least two independent experiments conducted in duplicate. Well diameter (0.6 cm) was subtracted from the measures.
